# Large-scale purification of functional AAV particles packaging the full genome using short-term ultracentrifugation with a zonal rotor

**DOI:** 10.1038/s41434-023-00398-x

**Published:** 2023-03-28

**Authors:** Mikako Wada, Naoya Uchida, Guillermo Posadas-Herrera, Hiromi Hayashita-Kinoh, Yuji Tsunekawa, Yukihiko Hirai, Takashi Okada

**Affiliations:** Division of Molecular and Medical Genetics, Center for Gene and Cell Therapy, The Institute of Medical Science, The University of Tokyo, Tokyo, Japan

**Keywords:** Gene therapy, Genetic transduction

## Abstract

Adeno-associated virus (AAV) vector-based gene therapy is potentially curative for various genetic diseases; however, the development of a scalable purification method for full-genome AAV vectors remains crucial to increase productivity and reduce cost of GMP production. In this study, we developed a large-scale short-term purification method for functional full-genome AAV particles by using 2-step cesium chloride (CsCl) density-gradient ultracentrifugation with a zonal rotor. The 2-step CsCl method with a zonal rotor improves separation between empty and full-genome AAV particles, reducing the ultracentrifugation time (4–5 h) and increasing the AAV volume for purification. The highly purified full-genome AAV particles were confirmed by analytical ultracentrifugation (AUC), droplet digital PCR (ddPCR) in the whole region of the AAV vector genome, transduction efficiency in target cells, and transmission electronic microscopy (TEM). The high-purity AAV9 particles were obtained using culture supernatant during vector preparation rather than cell lysate. CsCl could be simply removed by a hydroxyapatite column. Interestingly, ddPCR analysis revealed that “empty” AAV particles contain small fragments of the inverted terminal repeat (ITR), probably due to unexpected packaging of Rep-mediated ITR fragments. This large-scale functional AAV vector purification with ultracentrifugation would be effective for gene therapy.

## Introduction

Adeno-associated virus (AAV) vectors that express therapeutic gene products have shown great promise for gene therapy. Recently, AAV vector-based gene therapy trials have been reported in various hereditary diseases, including Duchenne muscular dystrophy (DMD), X-linked myotubular myopathy (XLMTM), hemophilia A, and hemophilia B [1, 2]. However, in DMD and XLMTM gene therapy trials, systemic injection of high-dose AAV9 vectors resulted in lethal liver failure at an early phase and death, most likely due to innate immunoreaction against the AAV genome and complement activation with AAV particles [1, 3–5]. In gene therapy in hemophilia A and hemophilia B using AAV2, AAV8, and AAV10 vectors, liver enzyme elevation and AAV capsid-specific T-cell activation were detected with subsequent declines in factor VIII and factor IX activity, respectively [2, 6].

To date, manufacturing purification methods for AAV vectors are generally based on ion-exchange and affinity chromatography [7] and this process can remove host cell proteins (HCPs). Recently, AAV8 and AAV9 serotypes have been more commonly used because of their higher efficiency gene delivery compared to AAV2, enabling the harvesting of AAV vectors from cell culture supernatant instead of cell lysate [8]. Moreover, these purified AAV vectors still vary according to the packaged genome sizes, including full-genome, intermediate, and empty particles, which are produced during the AAV biomanufacturing process [4]. Empty capsids are thought to reduce transduction efficiency and induce unnecessary immune responses. In addition, double-stranded RNA (dsRNA) can be generated by bidirectional promoter activity from the inverted terminal repeat (ITR) of AAV, enhancing innate immunity [9]. Ultracentrifugation with a density gradient of cesium chloride (CsCl) or iodixanol allows AAV vectors to more efficiently separate the full-genome and empty particles compared to chromatography [10–13]. However, this system is limited by its small scale, and long exposure to CsCl (conventionally for 2 days) reduces the transduction efficiency of AAV vectors [5, 14]. In addition, iodixanol is not suitable for clinical use because of its cross-reactivity with iodine allergy.

For short-term purification of full-genome AAV vectors, we previously developed a 2-step CsCl density-gradient ultracentrifugation method; however, this is limited to a small scale (180 mL). Therefore, in this study, we developed a large-scale (1000 mL), short-term purification system for functional full-genome AAV vectors using ultracentrifugation. In this system, a zonal rotor (1.7 L capacity) was used to increase the AAV vector loading volume during ultracentrifugation, and a 2-step CsCl density gradient in the zonal rotor allowed for faster separation of full-genome particles, resulting in shorter exposure to CsCl during ultracentrifugation and efficient recovery of full-genome AAV vectors.

## Materials and methods

### Preparation of AAV vectors

AAV vectors were prepared in a large scale and harvested from culture supernatant (conditioned media), as previously described [3]. In brief, a 293EB cell line expressing adenoviral *E1a*, adenoviral *E1b*, and *Bcl-x*_*L*_ [15] was expanded in two 500 mL flasks (HYPER*Flask*, Corning, Corning, NY, USA) or a 1 L bioreactor (iCELLis Nano Bioreactor, Pall, Port Washington, NY, USA) for 5 days or 4 days, respectively, in Dulbecco’s Modified Eagle Medium (DMEM high glucose, FUJIFILM Wako, Chuo-ku, Osaka, Japan) with 10% fetal bovine serum (Thermo Fisher, Waltham, MA, USA). Transfection was then performed with polyethylenimine max, (Polysciences, Warrington, PA, USA) using pAAV-ZsGreen1 (TaKaRa Bio, Kusatsu, Shiga, Japan), pRC9 (serotype 9), and helper plasmids in DMEM including 2 mM L-Alanyl-L-glutamine Solution(100x) (Nacalai Tesque, Nakagyo-ku, Kyoto, Japan), 0.12% NaHCO_3_ (Nacalai Tesque), and 0.13% D-glucose (Nacalai Tesque) without serum. Five days post-transfection, culture supernatants were harvested and treated with 18.5 U/mL endonuclease (KANEKA CORPORATION, Minato-ku, Tokyo, Japan) with 5 mM MgCl_2_ (Nacalai Tesque) for 30 min at 37 °C. All cells were checked for mycoplasma contaminations resulting were reported negative.

### Ultracentrifugation of AAV vectors with a zonal rotor

Five percent CsCl (FUJIFILM Wako) in HNE buffer (50 mM 4-(2-hydroxyethyl)-1-piperazineethanesulfonic acid (HEPES, FUJIFILM Wako), 0.15 M NaCl (Nacalai Tesque), and 25 mM ethylenediaminetetraacetic acid (EDTA, Nacalai Tesque), pH7.4) or HN buffer (50 mM HEPES and 0.15 M NaCl, pH7.4) were added to the culture supernatant, including AAV vectors (Table 1). A zonal rotor consists of a large cylindrical chamber subdivided into four sector-shaped compartments by vertical septa that radiate from the axial core to rotor wall. The entire chamber was used during centrifugation and loaded with a single density gradient, and each sector-shaped compartment served as a large centrifuge tube. The large chamber capacity of these rotors (1.7 L) eliminates the need for multiple runs and density gradients. A CsCl density gradient was generated in a zonal rotor (P32CT or P35ZT, Eppendorf Himac Technologies, Hitachinaka, Ibaraki, Japan) at 3000 rpm by loaded 200 mL HNE or HN buffer, AAV vector containing 5% CsCl, 300 mL of 25–27% CsCl in HNE or HN buffer, and 300 mL of 38–40% CsCl in HNE or HN buffer. AAV vectors were separated by ultracentrifugation (Himac CP 80NX, Eppendorf Himac Technologies) at 30,000–35,000 rpm for 4–10 h. After separation, 2 L of 42–45% CsCl buffer was slowly added to the inside of the zonal rotor at 3000 rpm, and each fraction within the zonal rotor was pushed out from the outside (Tables 2, 3). RI were measured in each fraction using an refractometer NAR-1T LIQUID or RX 5000i (Atago, Minato-ku, Tokyo, Japan). Each fraction sample was dialyzed with 20 kDa molecular weight cut-off dialysis cassettes (#66003 Thermo Fisher) in 0.5 mM MgCl_2_ (Nacalai Tesque) in water for ~2 h at 4 °C, and 0.5 mM MgCl_2_ in PBS (#27575–31, Nacalai Tesque) overnight at 4 °C.

**Table 1:** Conditions of zonal ultracentrifugation.

Experiment’s IDCondition	#Z1		#Z3		#Z5		#Z7	
Rotor	P32ZT		P32ZT		P35ZT		P35ZT	
rpm	30,000		30,000		35,000		35,000	
Duration of centrifugation	10 h		5 h		4 h		4 h	
Vector type	AAV9-GFP		AAV9-ZsGreen1		AAV9-ZsGreen1		AAV9-ZsGreen1	
	HNE buffer	300 ml	HNE	200 ml	HN	200 ml	HN	300 ml
	AAV (5%CsCl)	300 ml	AAV (5%CsCl)	900 ml	AAV (5%CsCl)	900 ml	AAV (5%CsCl)	1 L
	15%CsCl	300 ml	25%CsCl	300 ml	25%CsCl	300 ml	27%CsCl	300 ml
	25%CsCl	300 ml	40%CsCl	300 ml	40%CsCl	300 ml	38%CsCl	200 ml
	33%CsCl	500 ml						
	40%CsCl	200 ml

**Table 2:** RI and volume of each fraction in experiment #Z1.

Fraction	Volume (mL)	#Z1
1	250	1.346
2	250	1.349
3	50	1.352
4	50	1.353
5	50	1.354
6	50	1.355
7	50	1.357
8	50	1.358
9	50	1.359
10	50	1.360
11	50	1.360
12	50	1.361
13	50	1.363
14	50	1.363
15	50	1.365
16	50	1.365
17	50	1.366
18	50	1.367
19	50	1.368
20	50	1.369
21	50	1.369
22	50	1.370
23	50	1.371
24	50	1.372
25	50	1.372
26	50	1.373
27	50	1.375
28	50	1.376
29	50	1.377
30	50	1.378
31	50	1.378
32	50	1.379
33	50	1.379
34	50	1.380
35	50	1.380

**Table 3:** RI and volume of each fraction in experiments #Z3, #Z5, #Z7.

Fraction no.	Volume(mL)	#Z3	#Z5	#Z7
1	250	1.342	1.337	1.336
2	250	1.341	1.338	1.339
3	250	1.342	1.339	1.340
4	250	1.345	1.343	1.342
5	150	1.351	1.351	1.347
6	150	1.357	1.357	1.353
7	30	1.360	1.361	1.356
8	30	1.362	1.362	1.358
9	30	1.362	1.364	1.359
10	30	1.363	1.365	1.360
11	30	1.363	1.366	1.361
12	30	1.365	1.367	1.362
13	30	1.366	1.368	1.363
14	30	1.367	1.369	1.364
15	30	1.368	1.370	1.365
16	30	1.369	1.371	1.366
17	30	1.370	1.372	1.367
18	30	1.370	1.374	1.368
19	30	1.371	1.376	1.370
20	30	1.372	1.377	1.371
21	30	1.373	1.379	1.373
22	30	1.374	1.380	1.374
23	30	1.375	1.382	1.375
24	30	1.376	1.382	1.376
25	30	1.377	1.383	1.377
26	30	1.378	1.383	1.377
27	30	1.379	1.383	1.377
28	30	1.379	1.383	1.377
29	30	1.380	1.383	1.377

### Evaluation of genome copies, capsid proteins, and transduction efficiency of AAV vectors by quantitative polymerase chain reaction (qPCR), western blotting, and flow cytometry

After ultracentrifugation with a zonal rotor, AAV genome copies of each fraction were evaluated using the AAVpro Titration Kit (for Real Time PCR) Ver.2 (TaKaRa Bio, Kusatsu, Shiga, Japan) in a QuantStudio 3 Real-Time PCR System (Applied Biosystems, Waltham, MA, USA). The sample size was *n* = 3 minimally needed for statistically significance.

The AAV capsid proteins in each fraction were evaluated by western blot analysis. The samples were degraded with NuPAGE LDS sample buffer (Thermo Fisher) and NuPAGE Reducing Agent (Thermo Fisher), electrophoresed on a 4–15% (v/v) gradient polyacrylamide gel (Criterion TG Precast Gels, Bio-Rad, Hercules, CA, USA) with SDS running buffer (Nacalai Tesque), transferred to a PVDF membrane (Trans-Blot Turbo Midi PVDF Transfer Packs, Bio-Rad), and detected using anti-AAV VP1/VP2/VP3 mouse antibody (clone B1, Progen, Heidelberg, Germany) and Amersham ECL Mouse IgG, HRP-linked whole Ab (Cytiva, Marlborough, MA, USA)^5^.

Transduction efficiency was evaluated using ZsGreen1-positive percentages (%ZsGreen1) in the transduced 293EB cells. 293EB cells (1 × 10^5^ cells) were cultured in 24-well plates overnight and transduced with each sample fraction (300 μL per well) in serum-free DMEM containing 2 mM L-glutamine, 12.1% NaHCO_3_, and 12.9% D-glucose (300 μL per well). The next day, 600 μL of the same culture medium was added to each well, and %ZsGreen1 was evaluated by flow cytometry (FACSMelody, Becton Dickinson, Franklin Lakes, NJ, USA) at 3 days post-transduction and analyzed using FlowJo Version 7.1 (Becton Dickinson).

### Evaluation of full-genome and empty AAV particles by AUC

The purity of AAV vectors was analyzed using a Proteome Lab XL-I ultracentrifuge (Beckman Coulter, Indianapolis, IN, USA). Bulk AAV vector samples (400 μL) were applied to the Centerpiece on Cell Housing, and three cell houses with samples and one counterbalance were inserted into an AUC rotor. After equilibrating to 20 °C, samples were ultracentrifuged at 12,000 rpm at 20 °C, and the absorbance (260 nm) and interference were measured at 92 timepoints for 4–5 h. The percentages of full-genome, intermediate, and empty AAV particles were analyzed using SEDFIT (National Institutes of Health, Bethesda, MD, USA) [16] and visualized using GUSSI (UT Southwestern Medical Center, Dallas, TX, USA).

### Evaluation of whole genome regions packaged in AAV vectors by droplet digital PCR (ddPCR)

Whole regions of the AAV vector genome were evaluated in each fraction of the samples from ultracentrifugation with a zonal rotor using ddPCR. 1.1 μL of sample less than 10,000 copies/µL (total 22 μL) was mixed with target primer/probe mixes (ddPCR Copy Number Assy, BioRad) (Table 4); 900 nM primers and 250 nM probe in droplets containing these materials were generated by an Automated Droplet Generator (BioRad) followed by PCR reactions in a C1000 Touch Thermal Cycler (BioRad). A QX200 droplet reader (Bio-Rad) using the QuantaSoft software package (Bio-Rad) was used to detect fluorescent signals in each droplet.

**Table 4:** A list of probe/primer mixture.

Probe/primer numbers	Regions
dCNS114927258	Plasmid backbone – 5’ITR
dCNS186312283	5’ITR - CMV promoter
dCNS461612055	CMV promoter #1
dCNS288475852	CMV promoter #2
dCNS979672264	CMV promoter – Beta-globin intron
dCNS805699613	Beta-globin intron #2
dCNS412100847	Beta-globin intron #1
dCNS188296634	Beta-globin intron – ZsGreen1
dCNS683934997	ZsGreen1 #1
dCNS126933432	ZsGreen1 #2
dCNS121835080	ZsGreen1 #3
dCNS626366336	ZsGreen1 #4
dCNS568970821	ZsGreen1 #5
dCNS357921241	ZsGreen1 – hGH polyA
dCNS864495584	hGH polyA #1
dCNS975683196	hGH polyA #2
dCNS435517654	hGH polyA – 3’ITR #1
dCNS953120020	hGH polyA – 3’ITR #2
dCNS787498924	3’ITR – plasmid backbone
dCNS227817477	Ampicillin resistant gene #1
dCNS770986825	Ampicillin resistant gene #2
dCNS442935619	Plasmid Backbone #2

### Morphological analysis of AAV vectors by transmission electron microscopy (TEM)

Collodion membranes (Nissin EM, Shinjuku, Tokyo, Japan) were hydrophilized using an ion bombarder (Nisshin EM Co., type PIB-10), and 3 μL of AAV samples were placed to a hydrophilized grid for 1 min. After three times washing with 3 μL water, samples were stained with phosphotungstic acid (PTA) for 10 s. The samples loaded onto the membrane were analyzed using TEM (HT7800, Hitachi High-Tech, Minato-ku, Tokyo, Japan).

### Polishing of AAV vectors by hydroxyapatite column

Chromatography was performed using an ÄKTA avant 25 system (Cytiva, Marlborough, MA, USA) with a SuperloopTM 150 mL at a flow rate of 1.0 mL/min. A column (4.6 × 35 mm, Sugiyama Shoji Co., Ltd. Kanagawa, Japan) packed with CHT Ceramic Hydroxyapatite Type I, 40 m (Bio-Rad Laboratories Inc., Hercules, CA, USA) was equilibrated with 10 mM HEPES and 150 mM sodium chloride, pH 7.2. The samples were loaded onto the column and eluted with 50 mM sodium phosphate buffer and 150 mM sodium chloride at pH 7.2. The resulting eluate was monitored for ultraviolet (UV) absorbance at 260 and 280 nm and conductivity. The collected fractions were evaluated by qPCR, using primers and probes targeting ZsGreen1.

### Data analysis

All values are expressed as means ± SEM. Statistical analysis of the data was conducted using a one-way ANOVA. For all statistical analyses, significance was defined as *P*  < 0.01.

## Results

### Separation of large-scale AAV vectors among full-genome, intermediate, and empty particles by density-gradient ultracentrifugation in a zonal rotor

**Fig. 1:**
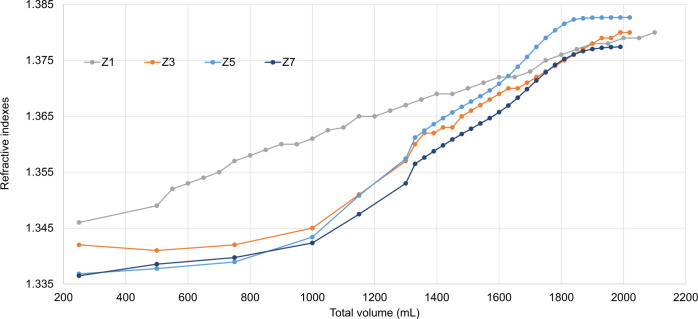
Adeno-associated virus (AAV) vectors were separated by density-gradient ultracentrifugation in a zonal rotor. AAV vectors were prepared in large-scale and harvested from the culture supernatant. Large amounts of AAV vectors were separated and fractionated by ultracentrifugation in a zonal rotor with serial (#Z1) or 2-step (#Z2–7) cesium chloride (CsCl) density-gradient. Each fraction was analyzed to measure the RI.

**Fig. 2:**
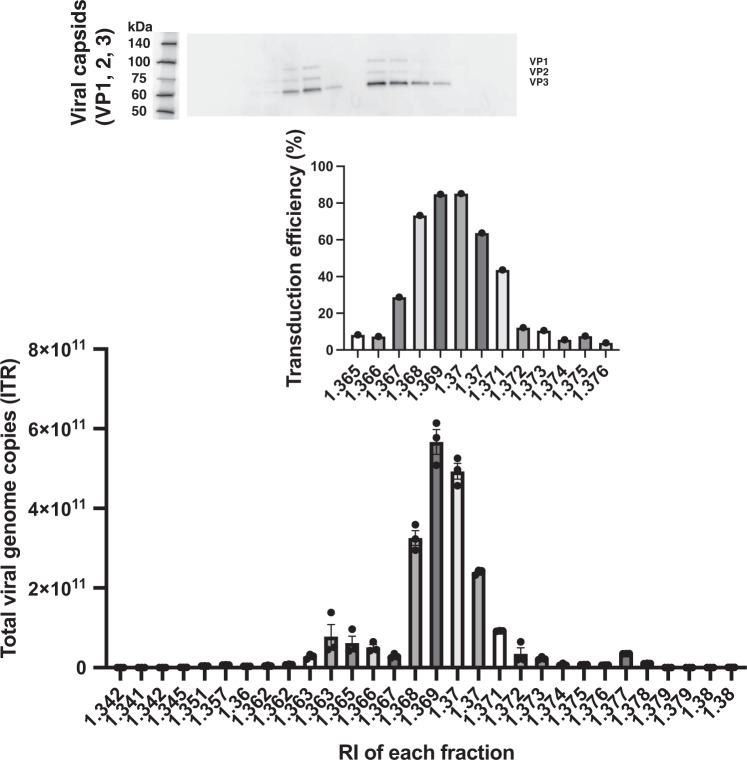
AAV vectors separated for full-genome, intermediate, and empty particles were analyzed by genome copies, transduction efficiency, and AAV capsid proteins. (Top) AAV capsids were detected by western blotting using anti-VP1 (82 kDa), VP2 (67 kDa), and VP3 (60 kDa) antibodies. (Middle) AAV transduction efficiency was evaluated using ZsGreen1 expression in transduced 293EB cells (*n* = 1, means ± SEM). (Bottom) AAV genome copies were measured by quantitative polymerase chain reaction (qPCR) using inverted terminal repeat (ITR)-targeting primers (*n* = 3, means ± SEM). Experiments were repeated six times in a single run.

We previously demonstrated a small-scale short-term purification method (180 mL, 2 h) for AAV vector fractions in 2-step CsCl density-gradient ultracentrifugation and Tangential flow-filtration, to remove contaminant HCPs and residual DNA [10]. To increase the amounts of AAV vectors in a short-term separation among full-genome, intermediate, and empty particles, this 2-step CsCl density-gradient was adapted to zonal rotor-mediated ultracentrifugation (Table 1). AAV vector-containing culture supernatant (inside) and 2–4 escalating densities of CsCl solutions (outside) were separately placed within a zonal rotor during low-speed centrifugation (3000 rpm), and the centrifuge speed was increased (30,000–35,000 rpm), allowing for density-gradient separation of large-scale AAV vectors (300–1000 mL). First, we performed four-step CsCl density gradient (15%, 25%, 33%, and 40%) ultracentrifugation using 300 mL AAV vector-containing solution for 10 h (Supplementary Fig. 1 and Table 2), resulting in a nearly linear CsCl gradient detected by refractive indexes (RI) among various fractions (Fig. 1 and Table 2). AAV capsid proteins were detected in two fractions of RI 1.366–1.367 and RI 1.368–1.371, and AAV genome copies and ZsGreen1 transduction efficiency (biological activity) peaked at the RI 1.368–1.371 fraction, demonstrating a separation between non-functional empty particles (RI 1.366–1.367) and functional full-genome particles (RI 1.368–1.371) (Fig. 2 and Table 3). Interestingly, the empty fraction contained some ITR (Fig. 2). Intermediate particles should be included between empty and full fractions.

The four-step density-gradient allowed for separation among full-genome, intermediate, and empty AAV particles; however, 10-hour CsCl exposure could reduce the biological activity of AAV vectors [5]. Therefore, we hypothesized that two-step densities of CsCl solutions instead of four-step can generate a small-range density-gradient focusing on AAV fractions, allowing for reduction of centrifugation time and an increase in the rotor space for AAV vectors instead of CsCl solutions (Table 1). Strikingly, two-step CsCl density-gradient ultracentrifugation (25–27% and 40%) could be completed with a shorter centrifugation time (4–5 h) as well as a larger volume of AAV vectors (900–1000 mL). The RI (density gradient) was more sharply elevated at a narrower range inside the zonal rotor (used for vector separation) and remained at a low level outside the zonal rotor (allowing for faster separation) (Fig. 1 and Table 3).

### High-purity full-genome AAV particles detected by AUC and transmission electron microscopy (TEM)

**Fig. 3:**
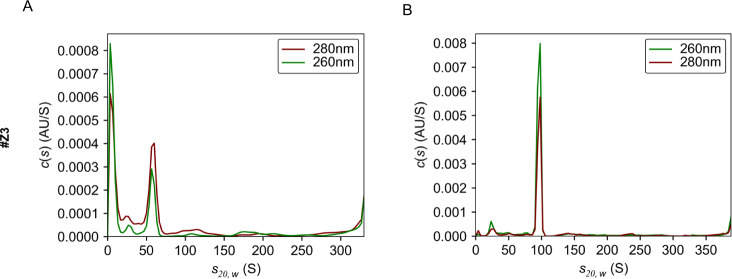
High-purity AAV vectors were separated from the culture supernatant by zonal ultracentrifugation. Overlays of distribution plot of the sedimentation coefficient of the data with absorbances at 260 nm and 280 nm by using empty (**A**, RI1.367) and full genome fractions (**B**, RI1.369) AUC experiments.

We performed AUC to evaluate the purity of the full genome (RI 1.370) and empty fraction (RI 1.368) [17], which were separated by two-step density-gradient ultracentrifugation with zonal rotor. We detected a single peak of AUC signals (70 ± 2%) with separate sedimentation coefficients between empty (approximately 60 S) (Fig. 3A) and the full genome (approximately 90 S) particles (Fig. 3B), demonstrating the high purity of AAV separation.

**Fig. 4:**
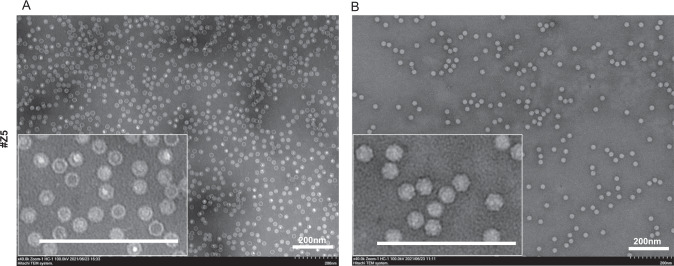
Morphology of full-genome and empty rAAV particles. rAAV particles in both empty (**A**, RI1.365) and full-genome (**B**, RI1.368) fractions were evaluated by negative staining in TEM.

We then morphologically analyzed the full-genome and empty AAV fractions using TEM with phosphotungstic acid (PTA) stain, which were separated by two-step density-gradient ultracentrifugation with a zonal rotor. In empty AAV fractions, a black dot was mostly observed in the center of the hexagon particles (Fig. 4A), whereas full-genome AAV fractions were detected as hexagon particles with light-density contents (Fig. 4B), suggesting that empty AAV particles might partially shrink and are more strongly stained by PTA.

### Inclusion of ITR fragments in the empty fraction of AAV vectors (low sedimentation coefficient fraction), evaluated by droplet digital PCR (ddPCR)

**Fig. 5:**
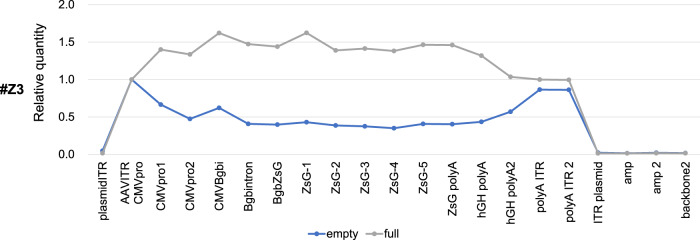
ITR fragments were contained in empty AAV particles, evaluated by droplet digital PCR (ddPCR). Various primer sets were designed to cover a whole region of the AAV genome, and the genome regions packaged in full-genome, intermediate, and empty AAV particle were evaluated by ddPCR.

To investigate which DNA fragments were packaged into full and empty AAV particles, 22 probe/primer sets were designed to detect the whole region of the AAV genome as well as the plasmid backbone (Fig. 5 and Table 4), as evaluated by ddPCR. We confirmed that the full-genome AAV particles (RI 1.369) contained the whole area of the AAV genome, along with slightly lower signals in the ITR area (Fig. 5). Interestingly, small ITR regions were detected in empty particles (RI 1.367).

### Polish of full-genome AAV vectors after ultracentrifugation with a hydroxyapatite column

**Fig. 6:**
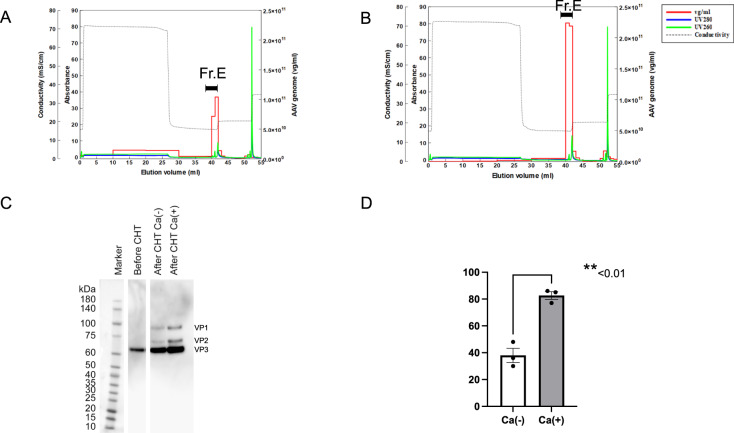
Polish of full-genome AAV vectors with a hydroxyapatite column. Full-genome AAV vectors (#Z7) were attached to a hydroxyapatite column and eluted by sodium phosphate. UV absorbance at 260 nm and 280 nm, and viral genome titer of each fraction of chromatography without (**A**) or with CaCl_2_ (**B**) were evaluated. AAV genome (vg/ml), UV absorbance at 260 nm, UV absorbance at 280 nm and the conductivity of elution buffer were measured. Capsids were detected by western blotting with anti-VP1, VP2, and VP3 antibodies (**C**). **D** Recovery of rAAVs (Fr.E) polished by CHT in all fractions was determined by viral genome titer (*n* = 3, means ± SEM).

Ceramic hydroxyapatite (CHT) has been successfully used to separate viral vectors [18]. To remove CsCl from purified AAV vector fractions, we developed a polishing method for full-genome AAV particles using hydroxyapatite column chromatography. Full-genome AAV particles were attached to hydroxyapatite and eluted with density-escalating phosphate buffers. We observed different elution peaks of full-genome AAV particles, contaminants including proteins, and dsDNA from the culture medium and host cell proteins (HCPs), allowing for the purification of the full-genome AAV particles (Fig. 6A, B). The rAAVs polished using CHT were then concentrated (Fig. 6C).

Several experiments were performed to increase the AAV9 vector-binding capacity of CHT resins. One key improvement to the function of the CHT resins was obtained by the addition of Ca^2+^ [19]. When the solution lacked calcium ions, the vectors were detected in the flow-through fraction (Fig. 6A). The yield loss was approximately 70%, and the addition of CaCl_2_ increased the recovery ratios of AAV particles to approximately 85% (Fig. 6B, D). These data demonstrate that CsCl can be easily removed using a hydroxyapatite column.

## Discussion

In this study, we developed a large-scale and short-term purification method for high-purity and full-length AAV vectors using two-step CsCl density-gradient ultracentrifugation with a zonal rotor (Fig. 1). This method allows for the reduction of ultracentrifugation time to 4–5 h and an increase in the AAV vector volume up to 1000 mL (Fig. 2). We confirmed that the high-purity separation of full-genome AAV particles by AUC and TEM (Figs. 3, 4), and small DNA fragments, including the ITR, were detected within empty particles by ddPCR (Fig. 5). The purification of functional full-genome AAV particles is preferred to improve the efficacy and safety of AAV vector-based gene therapy, owing to the removal of contaminant HCPs and residual DNA, as well as non-functional intermediate and empty AAV particles.

To date, capsid antibody-based affinity columns and/or anion-exchange columns are commonly used for AAV vector purification [20–23], since they are more scalable and well-established for clinical-grade production without complete separation among full-genome, intermediate, and empty AAV particles [11, 24]. In contrast, these AAV particles can be separately detected by AUC according to the variance of densities among AAV particles, and thereby, ultracentrifugation-based purification is theoretically preferable for purification of full-genome AAV particles [25, 26]. We have previously demonstrated lab-scale, short-term purification of full-genome AAV particles using a 2-step CsCl density gradient [11], and in this study, this method was utilized for zonal ultracentrifugation. The full-genome AAV vector particles were fractionated with zonal ultracentrifugation, and purity was confirmed by infectivity (Fig. 2), AUC (Fig. 3), and TEM images (Fig. 4). In this system, 1000 mL of AAV vectors can be applied for one-cycle ultracentrifugation, and a further increase in sample volume is preferable for clinical-grade production.

Surprisingly, ITR signals were detected in “empty AAV particles,” as analyzed using ddPCR (Fig. 5). In contrast, “full-genome AAV particles” contain whole regions of the DNA genome between two ITRs, along with slightly lower signals in the ITR regions. In our current understanding, AAV Rep recognizes and nicks a terminal resolution site close to both 5’ and 3’ ITRs, and it might generate not only 2-ITR full-genome but also 1-ITR full-genome and small ITR only fragments, which can be packaged into AAV capsids [27, 28]. Both 2-ITR and 1-ITR full-genome particles should express the transgene as functional vectors, but ITR-only “empty” particles should be non-functional. This may be a major reason why empty AAV particles are significantly generated in AAV vector preparation. Moreover, AAV genome-based titers are sometimes evaluated using ITR-specific primers; therefore, ITR-based AAV genome titers can be overestimated because of the inclusion of ITR-packaged empty particles. In gene therapy, the administration dose of AAV vectors is usually calculated by vector genome titer (v.g./mL); thus, accurate titrations of AAV vectors are important for clinical usage [29]. The promoter activity of ITR produces dsRNAs, most likely inducing innate immunity against AAV vectors. The accumulation of dsRNAs was reported to stimulate the MDA5 sensor in human hepatocytes after AAV vector transduction, leading to the expression of type I interferons [30, 31]. A low-dose administration of the AAV5 vector resulted in no elevation of liver enzymes, minimal T-cell activation, and sustained coagulation factor activity among most patients in a hemophilia gene therapy [9, 32]. These data suggest that high purification of full-genome AAV vectors can reduce a total dose of AAV vectors as well as the inclusion of small ITR fragments within empty particles and may prevent the immune activation in response to AAV vectors. Further evaluation of immunoreactions to full-genome and empty AAV particles is required.

In the process of AAV vector production, removal of CsCl is required for the clinical use of two-step CsCl density gradient zonal ultracentrifugation. In this study, we developed a buffer-change method to remove CsCl using a CHT and adding calcium ion allowed higher recovery of AAV vectors (Fig. 6). This method should be applicable to purify large-volume AAV vectors, and the elution buffer in this method appears to be milder than the solution used in chromatography at low pH. This system can be used to polish AAV vectors to remove HCPs with minimal damage following ultracentrifugation (Supplementary Fig. 2).

In addition, we purified AAV2 vectors using the two-step CsCl density-gradient ultracentrifugation with a zonal rotor (Supplementary Fig. 3). AAV2 vectors were collected from cell pellets, followed by affinity chromatography before zonal ultracentrifugation. It yielded about 1.0 × 10^13^ v.g of full-genome particles, which are similar to that of AAV9 vectors collected from the culture supernatant. These data suggest that zonal ultracentrifuge could be applicable to various serotypes.

In summary, we developed a large-scale, short-term purification method for full-genome AAV vectors using two-step CsCl density gradient ultracentrifugation with a zonal rotor. High-purity full-genome particles were confirmed using genome copies per capsid protein, ddPCR signals in the whole AAV genome, ZsGreen1 expression, AUC, and TEM images. We also demonstrated that empty AAV particles contain small ITR fragments, possibly because the Rep-mediated nick generates ITR fragments, resulting in packaging of AAV capsids. Moreover, polishing of AAV vectors with CHT successfully concentrated AAV vectors and remove HCPs and CsCl. This large-scale AAV purification system can be effective in clinical research such as GMP manufacturing.

## Supplementary information

Supplemental figures

## Data Availability

The datasets generated during and/or analyzed during the current study are available from the corresponding author on reasonable request.
