# Supplementary material for: Large-scale purification of functional AAV particles packaging the full genome using short-term ultracentrifugation with a zonal rotor
Source: Gene Ther. 2023 Mar 28;30(7-8):641–8. doi: 10.1038/s41434-023-00398-x (PMC10457186; doi:10.1038/s41434-023-00398-x)
Supplement: Supplementary file 1 — Supplemental figures [file 41434_2023_398_MOESM1_ESM.pdf]

Supplementary Figure1

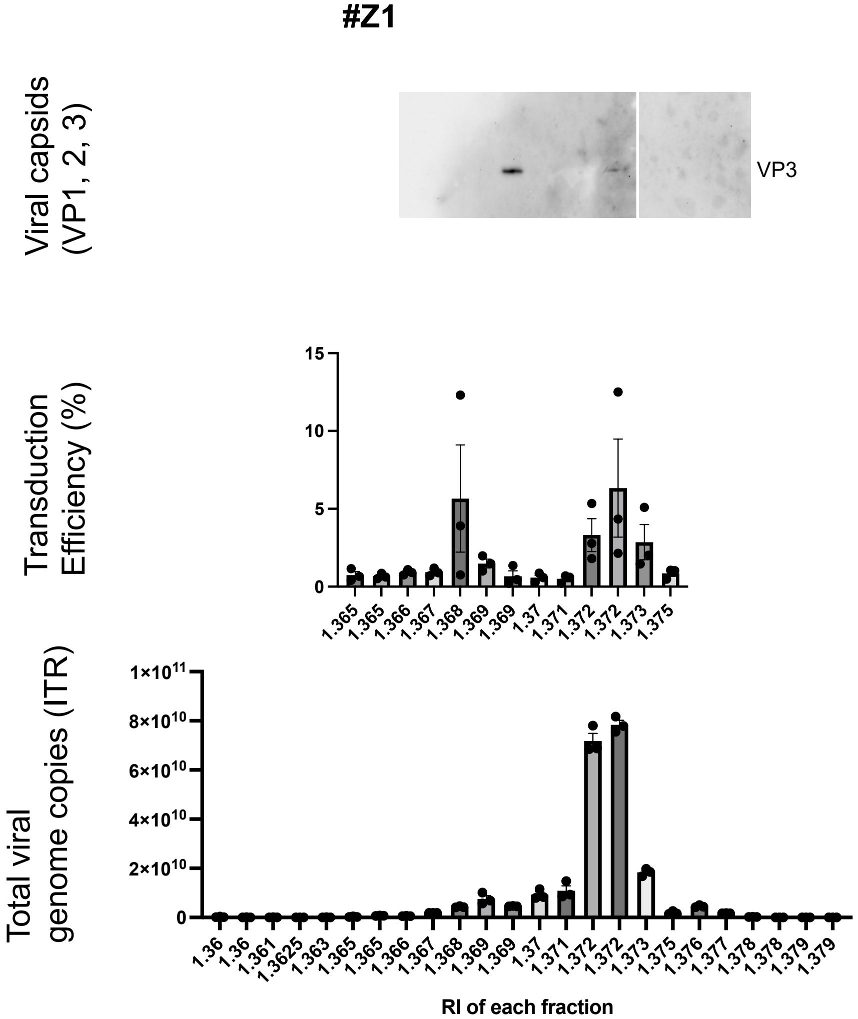

Supplementary Figure 1. AAV vectors purified with four-step CsCl density-gradient ultracentrifugation separated for full-genome, intermediate, and empty particles were analyzed by genome copies, transduction efficiency, and AAV capsid proteins. (Top) AAV capsids were detected by western blotting using anti-VP1 (82kDa), VP2 (67kDa), and VP3 (60kDa) antibodies. (Middle) AAV transduction efficiency was evaluated using ZsGreen1 expression in transduced 293EB cells (n=3, means±SEM). (Bottom) AAV genome copies were measured by quantitative polymerase chain reaction (qPCR) using inverted terminal repeat (ITR)-targeting primers (n=3, means±SEM).

Supplementary Figure2

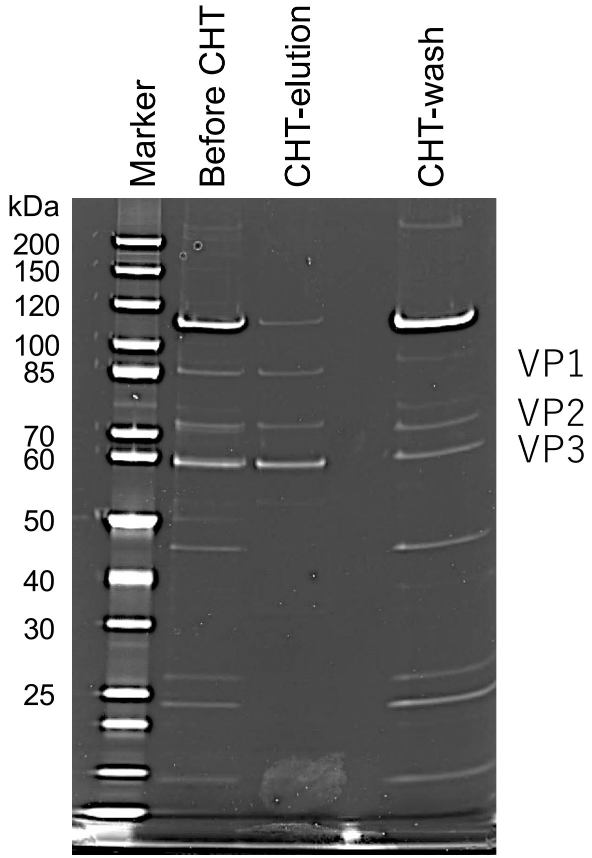

Supplementary Figure 2. Polishing of AAV vectors with CHT remove HCPs.
Each step of polishing by a hydroxyapatite column was analyzed by 4–15% (v/v) gradient gel SDS-PAGE and Oriole fluorescent gel stain.

Supplementary Figure3

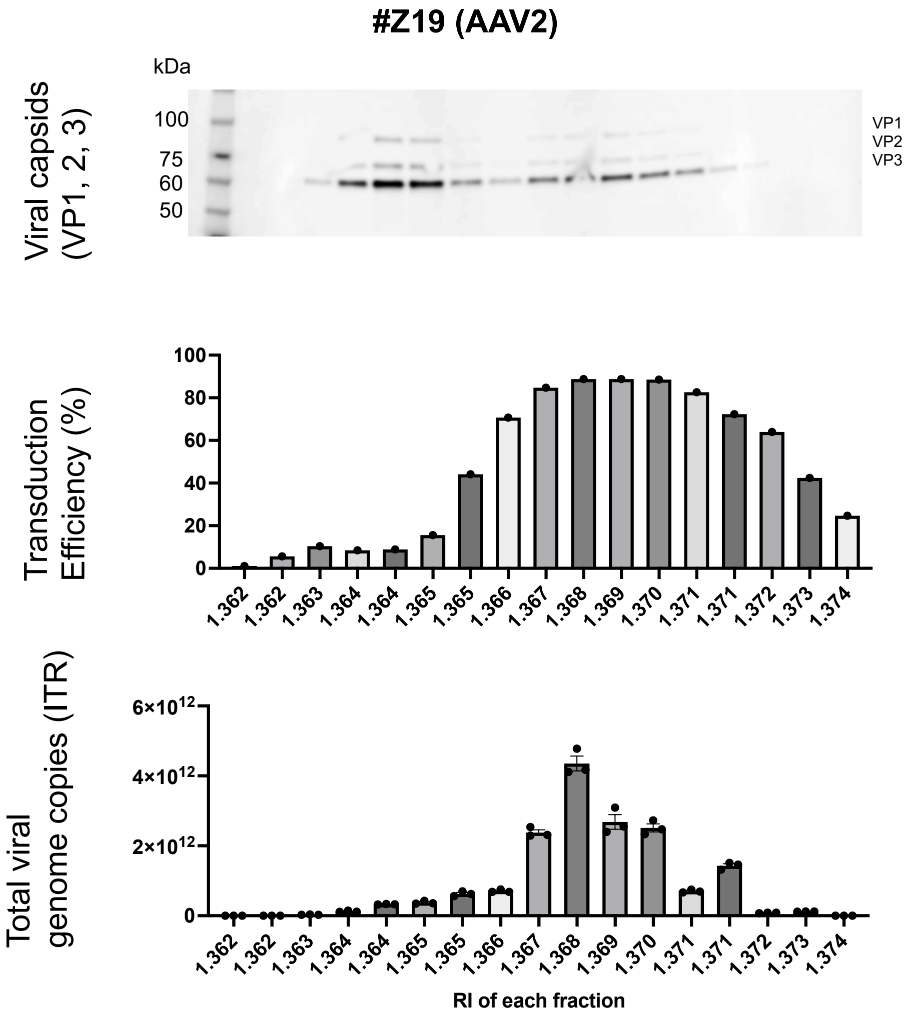

Supplementary Figure 3. AAV vectors purified with two-step CsCl density-gradient ultracentrifugation separated for full-genome, intermediate, and empty particles were analyzed by genome copies, transduction efficiency, and AAV capsid proteins. (Top) AAV capsids were detected by western blotting using anti-VP1 (82kDa), VP2 (67kDa), and VP3 (60kDa) antibodies. (Middle) AAV transduction efficiency was evaluated using ZsGreen1 expression in transduced 293EB cells (n=1, means±SEM). (Bottom) AAV genome copies were measured by quantitative polymerase chain reaction (qPCR) using inverted terminal repeat (ITR)-targeting primers (n=3, means±SEM).
